# Embryos With “No Result” After PGT-A: A Retrospective Analysis of Causative Factors

**DOI:** 10.1155/ogi/4043963

**Published:** 2025-03-24

**Authors:** Anastasia Salame, Elias M. Dahdouh, Mokhamad Zhaffal, Rania Aljafari, Arya Muraleekrishnan, Aparna Bajpai, Shabin Kainoth, Leyla Depret Bixio, Michael Fakih

**Affiliations:** ^1^Department of Reproductive Endocrinology and Infertility, Fakih Fertility Centre, Al Ain, UAE; ^2^Department of Obstetrics and Gynecology, CHU Sainte-Justine, Montreal, Quebec, Canada; ^3^Division of Reproductive Endocrinology and Infertility, Université de Montréal, Montreal, Quebec, Canada; ^4^Department of Obstetrics and Gynaecology, Kanad Hospital, Al Ain, UAE

**Keywords:** blastocyst, preimplantation genetic testing for aneuploidy, trophectoderm biopsy, vitrification

## Abstract

**Background:** “No result” after PGT-A is a rare observation. Factors suspected to cause inconclusive diagnoses include poor embryo quality, day of biopsy, biopsy technique, and technical amplification failure due to diluted DNA material. This study aimed to highlight the predisposing factors that could lead to a “no result” observation after PGT-A.

**Results:** This is a retrospective cohort study involving 177 patients and 1335 blastocysts, 1242 of which comprised the control (result) group and 93 comprised the study (no result) group. The predisposing factors studied were the number of blastocysts available for biopsy, the day of biopsy, the grade of the embryo, the degree of expansion of the blastocyst, and the grade of the trophectoderm on biopsy day. The rate of “no result” embryos did not depend on the degree of expansion of the embryo, the trophectoderm quality, the day of biopsy, or embryo grade (*p*=0.139, 0.34, 0.332, and 0.272, respectively). Regression analysis showed that the studied embryo characteristics were not significant predisposing factors. However, having more blastocysts to biopsy per patient was found to be a significant predictor of “no result” embryos.

**Conclusion:** No clear embryo-related risk factors could be elucidated; however, the biopsy procedure and sample cellularity seem to be crucial components. In addition, having more embryos per patient to biopsy at a given time might increase the risk of having inconclusive biopsy results.

## 1. Introduction

Embryo biopsy for genetic testing has been on the rise since the 1990s. This is specifically used in preimplantation genetic testing for aneuploidy (PGT-A), monogenetic mutation (PGT-M), and chromosomal structural rearrangement (PGT-SR). Initially, cleavage-stage embryos were biopsied for the extraction of 1-2 blastomeres [1]. This practice was later replaced by Day 5-6 blastocyst-stage biopsy for the extraction of 5–10 trophectoderm (TE) cells, as it produced more accurate results and removed any detrimental effect on implantation rates [2–4]. Genetic testing on a transferrable embryo results in either euploid or aneuploid, the latter being not transferred in most cases. Notably, studies have reported a test failure rate of 2%–7% [4, 5]. Other known terms include nonconcurrent diagnosis, failed analysis, and no call or no result embryos (NR). This is usually thought to be due to an insufficient amount of DNA, low quality of DNA, DNA amplification failure, or failure to load the TE cells into the PCR tube, also known as tubing [6]. Other factors known for causing inconclusive diagnoses include poor-quality DNA secondary to poor embryo quality, day of biopsy, biopsy technique, shipping conditions, and technical amplification failure due to diluted DNA material [7]. However, exact predisposing factors for inconclusive analysis are poorly understood, and studies addressing the issue are scarce [7–9]. To avoid the wastage of NR embryos, warming, rebiopsy, and a second round of cryopreservation are usually undertaken. However, few studies highlight the effects of rebiopsy and revitrification on the reproductive potential of NR blastocysts. The findings ranged between completely unfavorable reproductive outcomes to equivalent outcomes when compared to single biopsy and single vitrification cycle embryos [4, 8, 10].

In our study, we aimed to study the effect of the grade, as well as the expansion of the blastocyst per se, and not just the grade of the embryo on the day of the biopsy on the incidence of failed testing.

## 2. Materials and Methods

**Figure 1:**
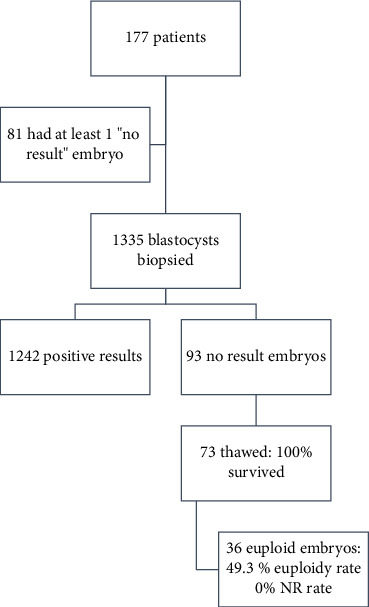
Plan of the study.

A retrospective analysis was carried out, and all patients undergoing ovarian stimulation cycles for PGT-A testing in a private practice in the United Arab Emirates between December 1, 2018, and December 31, 2021, were included. The reviewed charts accounted for 1335 blastocysts biopsied in the setting of PGT-A cycles, resulting from 177 patients who underwent controlled ovarian stimulation cycles. The predisposing factors included in the analyses were the day of the biopsy, the grade of the embryo, the degree of expansion of the blastocyst, and the grade of the TE on the biopsy day. The study plan is summarized in Figure 1.

The study was approved by the research ethical committee (REC) of the fertility center.

### 2.1. Description of Treatment Cycles and Genetic Testing

The patients underwent PGT-A for advanced reproductive age (ARA), severe male factor, consanguinity, recurrent pregnancy loss (RPL), recurrent implantation failure (RIF), gender selection, and personal choice. The controlled ovarian stimulation protocol used was the antagonist protocol. The type and the dose of the gonadotropins to be used were decided based on the baseline hormonal profile, antral follicular count, and ovarian response to stimulation. The type and dose of the ovulation trigger depended on the number of follicles on trigger day and the estrogen level based on previous publication by our group [11].

#### 2.1.1. Embryo Biopsy

Embryo biopsy was performed in accordance with the facility's internally validated standard operating procedure (SOP). The grading of the embryos on the day of the biopsy was performed based on the grading system adapted by Capalbo et al., where embryos graded as 3, 4, 5, 6 AA were labeled as excellent quality embryos, embryos graded as 3, 4, 5, 6 AB/BA were described as good quality embryos, blastocysts graded as 4, 5, 6 BB/AC/CA were described as average quality, and embryos with a grade of 3 BB/BC/CB were labeled as poor [12]. Using a laser (LYKOS-Hamilton Thorne) with 200 pulses, a hole measuring 7 microns was created in the zona pellucida during the cleavage stage on Day 3 embryos as part of assisted hatching. This was done based on the SOP followed at our fertility clinic during the study period. On Day 5, the well-expanded blastocyst with herniating TE from the zona breach was biopsied to get an ambient number of 6-7 cells using the pulling technique with additional low-pulse laser (LYKOS-Hamilton Thorne). The nonexpanded blastocysts were further cultured till Day 6 for adequate expansion. After the aspiration, the TE cells were washed twice in wash buffer (ORIGIO Handling IVF Medium), placed in 10 microliters of HEPES buffered drop of media, and then transferred into a post-biopsy four-well dish (ORIGIO Handling IVF Medium) labeled with the patient's information. The biopsied blastocysts were washed twice in culture media and then placed back in the corresponding drop of culture medium in a new culture dish, followed by cryopreservation using Cryotech Vitrification. In the event of no result embryos after the initial biopsy, the couples were counseled, consented, and then the blastocysts were thawed using the Cryotech warming kit; once embryo re-expansion was confirmed post-warming, the biopsy protocols and cryopreservation were repeated. The embryo biopsy was performed by 3 highly trained senior embryologists with double verification to ensure proper identification and strict adherence to the lab's SOP [11].

#### 2.1.2. Genetic Testing

The samples were transferred to the in-house genetic lab. The samples were loaded into PCR tubes (tubing) to start the DNA amplification process by 2 qualified senior staff. The number of the blastomeres ranged from 5 to 9 cells. The DNA was amplified using multiple displacement amplification (MDA). The MDA product was then processed for library preparation and sequencing by high-resolution next-generation sequencing (NGS-HR). The sequencing platform used was MiSeq from Illumina. Once the sequencing data were generated, they were transferred to a computer, and the results were viewed using the Bluefuse software [11].

### 2.2. Statistical Analysis

Statistical analysis was performed using IBM SPSS Statistics Version 29.0 (IBM, Chicago, IL, USA). Model assumptions for the continuous variables were checked using the D'Agostino and Pearson test and Shapiro–Wilk test. Whenever the model assumptions were violated, the difference between study groups (control and rebiopsy groups; control and no-result embryo groups) was determined by the nonparametric Mann–Whitney *U* test. Chi-square or Fisher's exact test was used for categorical variables. Continuous variables are presented as mean ± SD, and categorical variables are presented as percentages and counts. Binary logistic regression was used to determine the odds of a no-result biopsy and predisposing factors. All the variables were entered using a forced entry method, and all the predictor variables were tested in one block to assess their predictive ability while controlling for other predictors in the model. The results are presented as an adjusted odds ratio with 95% CI. A two-sided *p* value of 0.05 was considered statistically significant.

## 3. Results

Patients having at least 1 NR embryo accounted for 45.76% of the patients studied. Comparison between the 2 group of patients (those who did not have NR embryos and those having at least 1 NR embryo) is included in Table 1. The female age, male age, duration of infertility, and number of blastocysts available for biopsy were found to be significantly different between the 2 groups (30.5 ± 6.2 vs. 32.4 ± 5.9, *p* < 0.0001, 34.4 ± 8.3 vs. 35.4 ± 7.7, *p*=0.0235, 2.4 ± 2.5 vs. 2.8 ± 2.4, *p*=0.0027, and 6.4 ± 0.25 vs. 7.3 ± 0.3, *p*=0.0191, respectively). The regression analysis revealed that the number of blastocysts per patient increases the probability of having no results (OR: 1.130, 95% CI: 1.033–1.236, *p*=0.0076) (Table 2).

**Figure 2:**
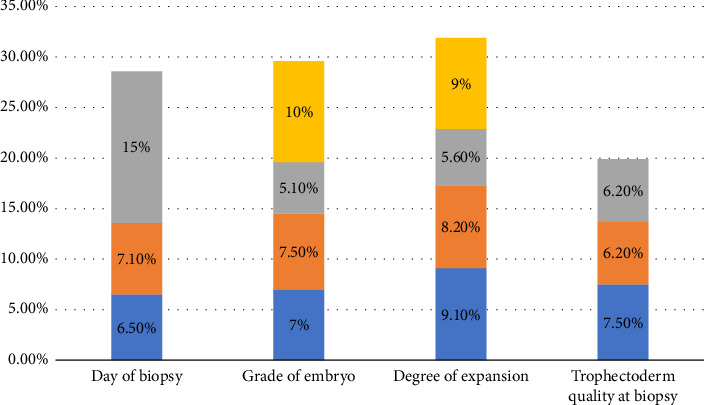
Incidence of no result embryos depending on the different variables studied. Day of biopsy coding: blue: Day 5, orange: Day 6, and gray: Day 7; *p* value: 0.3327. Grade of embryo: blue: excellent, orange: good, gray: average, and yellow: poor; *p* value: 0.272. Degree of expansion: blue: 3, orange: 4, gray: 5, and yellow: 6; *p* value: 0.1392. Trophectoderm quality: blue: A, orange: B, and gray: C; *p* value: 0.34.

Out of the 1335 blastocysts biopsied, 93 produced no result, that is, a rate of 7%, as presented in Figure 1. The demographic data were comparable between the two groups, as presented in Table 3. The number of blastocysts available for biopsy was significantly higher in the control group compared to the NR group (9.424 ± 5.05 vs. 7.753 ± 4.15, *p*=0.004) as presented in Supporting Table 1.  Table 4 presents the characteristics of the embryos subjected to biopsy in the control and the NR group. Concerning the degree of expansion of the embryo, the TE quality, day of biopsy, and embryo grade, there was no difference in the rate of NR embryos (*p*=0.139, 0.34, 0.332, and 0.272, respectively) (Figure 2). Regression analysis revealed that the day of the biopsy, grade at the biopsy, degree of expansion, and TE quality were not found to be statistically significant factors (*p*=0.71, 0.525, 0.484, and 0.32, respectively). However, the number of blastocysts from the cohort of all embryos available for biopsy was found to significantly decrease the incidence of NR embryo, with an OR of 0.923 and a 95% CI (0.86–0.99). The results are presented in Table 5.

## 4. Discussion

In the current retrospective analysis, we found that the incidence of inconclusive biopsies in our series was consistent with the published figures of 2.5%–7%, albeit being on the upper accepted limit [4, 5, 8]. Out of the analyzed risk factors for NR embryos, having a higher number of blastocysts available for biopsy was found to be a protective factor against inconclusive results. Interestingly, when patients were compared to each other, it was found that having more blastocysts for biopsy per patient increases the risk of having “no result” embryos. In other words, when the embryologist has more embryos to biopsy at a given time, the chances of having inconclusive results are higher highlighting a possible human factor predisposing to NR. However, from a training perspective, having more embryos over a certain period of time meant that the embryologists had more hands on perfecting the biospy technique and thus reducing significantly the NR rates over the long run.

Minimal data available on the topic and the main drawbacks of the different studies can be attributed to the lack of a standardized embryo grading system. Cimadomo et al. used Gardner's grading system which was adapted by Capalbo et al., in which embryos were categorized into excellent, good, average, and poor [8, 12]. Bradley et al. used the simplified Gardner grading system that divides the embryos into excellent, good, and poor, while De Vos et al. used a different grading system to divide the embryos into top, good, and moderate [4, 10]. The absence of a unified grading system made the assessment of the TE suboptimal before the biopsy procedures. This is of utmost importance since Cimadomo et al. postulated that the degree of expansion of the blastocyst might affect the incidence of the NR embryos. In their analysis, they found that Day 5 biopsies were associated with significantly higher rates of NR embryos when compared to Day 6 and Day 7 biopsies, with the rates being 3.7%, 2.1%, and 1.1%, respectively. The group postulated that the number of TE aspirated during the biopsy is inversely proportional to the incidence of NR embryos, with the best number of TE being eight cells taken on Day 6. The authors further suggested that on Day 6, blastocysts were supposed to have a better expansion when compared to Day 5 given that the assisted hatching was done on Day 5, and thus it became more feasible to aspirate more cells during the biopsy [8]. To test this theory, we included the actual degree of expansion of the blastocyst in the analysis of the causative factors. Our data showed that the rate of NR was equal between the different degrees of expansion (3 = 9.1%, 4 = 8.2%, 5 = 5.6%, and 6 = 9%; *p*=0.139). No difference was noted in the rate of NR embryos between embryos with TE grade *A*, *B*, or *C* (*A* = 7.5%, *B* = 6.2%, and *C* = 6.2%; *p*=0.34) (Figure 2). This only highlighted the fact that the quality of the TE did not correlate with the actual quantitative DNA content of the cell. Our findings were in contrast with Neal et al., who found that biopsied Grade B and C TE cells significantly increased the rate of NR embryos when compared to Grade A TE cells [7]. Neal et al. acknowledged that the cellularity of the biopsied samples was not studied, and as such it might be probable that in their series, the Grade B and C TE biopsied had erroneously fewer TE to analyze. A possible explanation for the differences noted between our study and Cimadomo et al.'s study is the day of the assisted hatching. In our study, it was on Day 3, while in the Italian group, it was on Day 5. Concerning the effect of the number of TE retrieved, we were not able to include the variable in our analysis as the genetic lab did not include the number of cells analyzed in the final report. However, since the tubing for PCR was done in the genetics lab, the number of cells ranged persistently between 6 and 9 as per internal validation reports of our lab.

All embryologists who performed biopsies at our lab are experienced and qualified in blastocyst biopsies. Osman et al. stated that embryologists' experience was an important factor in reducing the nonconclusive results [9]. This was supported by Cimadomo et al., who reported statistically significant different NR rates between six IVF centers; they also found the importance of embryologists' experience in obtaining enough cells [8]. Another study by Mizobe et al. compared the two biopsy techniques—pulling and flicking methods—for differences in PGT-A results. The genetic analysis outcomes in the study did not include the NR rate. However, the group concluded that the presence of at least five TE cells available for analysis was a significant predictor for the availability of genetic results. Other factors such as different operators, time taken for biopsy procedure, and laser irradiation were not found to be statistically significant. The group reported that the biopsies performed using the pulling method were found to have significantly fewer cells when compared with the flicking technique [13]. This is important, as a small number of extracted TE was considered a predisposing factor for NR embryos [8].

Given that the number of rebiopsied embryos transferred in the setting of frozen embryo transfer was low in our center, we decided to not include the reproductive outcomes. However, the literature revealed that the most recent studies by Cimadomo et al., Priner et al., and Taylor et al. agreed on the lack of a negative impact on the implantation potential. Priner et al. showed that there was no significant difference in clinical pregnancy and live birth rates when compared to embryos that had one successful biopsy, thereby supporting the findings of Taylor et al. [8, 14, 15]. Cimadomo et al. also showed favorable outcomes of transfer of rebiopsied embryos with comparable pregnancy rates, miscarriage rates, and live birth rates [8]. Zhang et al. also found that performing a rebiopsy negatively impacted neither the implantation potential nor the pregnancy rates [5]. On the other hand, Neal et al. showed a statistically significant reduced continuing pregnancy rate and a trend toward higher clinical loss after transfer of rebiopsied blastocysts; however, further subanalysis showed a possible negative impact of the multiple vitrification cycles rather than the rebiopsy itself [7]. Bradley et al. also showed a significant reduction in pregnancy outcomes when biopsy and vitrification-warming were performed twice, with the live birth rate decreasing from 50% in the single biopsy single vitrification warming cycle to 27.3% in the rebiopsy group. Further, chemical pregnancies increased without reaching statistical significance in the rebiopsy group (18% vs. 13%). In addition, neonatal outcomes in terms of gestational age at delivery and birth weight were found to be comparable. Interestingly, in the later study, the rate of poor embryos was significantly higher in the rebiopsy group (27.65%) than in the control group (8.7%), as well as the absence of Day 5 embryos in the rebiopsy group in comparison to 57.4% in the control group. These differences could explain the negative impact of rebiopsy. The same study also concluded that multiple vitrification cycles had minor nonsignificant effects on the implantation and developmental potential of the embryos [4]. This was supported by Oliva et al., who found that repeated vitrification cycles were not significant factors [16].

The strength of our study is the large sample size, where more than 1000 blastocysts were analyzed. To the best of our knowledge, it is the first study that has included the actual degree of expansion of the biopsied blastocysts in the analysis.

The main limitation of this study is its retrospective nature, as it increases the chances of bias. However, any other study design might not be ethically feasible.

## 5. Conclusion

We conclude that embryos producing “no result” after PGT-A are rare. No clear embryo-related risk factors could be elucidated. However, the biopsy procedure and sample cellularity seem to be crucial factors. In addition, having more embryo to biopsy at a given time might increase the risk of having inconclusive biopsy results highlighting the possible human factor in influencing the rate of “no result” embryos.

## Figures and Tables

**Table 1:** Descriptive analysis of the variables between the patients who had no “no result” embryos and the patients who had at least 1 “no result embryo.”

Characteristics	Combinations without no results *n* = 96	Combinations and no results *n* = 81	*p* value
Female age	30.5 ± 6.2	32.4 ± 5.9	< 0.0001
Male age	34.4 ± 8.3	35.4 ± 7.7	0.0235
Duration of infertility	2.4 ± 2.5	2.8 ± 2.4	0.0027
Number of OOC	19.1 ± 0.4	20.0 ± 0.5	0.1757
Number of MII	15.4 ± 0.4	15.7 ± 0.4	0.6047
Number of fertilized	13.0 ± 0.36	13.2 ± 0.4	0.7057
Number of blastocyst	6.4 ± 0.25	7.3 ± 0.3	0.0191
Duration of infertility	2.8 ± 0.3	2.8 ± 0.3	0.9422
Cause of infertility			0.6771
Female factor	15 (15.6)	13 (16.0)	
Male factor	39 (40.6)	27 (33.3)	
Mixed	29 (30.2)	31 (38.3)	
Unexplained	13 (13.5)	10 (12.3)	
Consanguinity			0.3418
None	66 (69.0)	51 (63.0)	
Yes	30 (31.0)	30 (37.0)	
Subdivision of infertility causes			0.9025
Abnormal semen analysis	69 (72.0)	60 (74.0)	
PCOS	13 (13.5)	15 (18.5)	
Low ovarian reserve	15 (15.6)	14 (17.3)	
No female cause	51 (53.1)	37 (45.7)	
Uterine factor	10 (10.4)	10 (12.3)	
Tubal factor	5 (5.2)	4 (5.0)	
Endometriosis	2 (2.1)	1 (1.2)	

**Table 2:** Regression analysis of the significant predictors between the 2 groups of patients (no “no result” embryo vs. at least 1 “no result” embryo).

Effect	Odds ratio	95% Wald confidence limits		*p* value
Age female	1.025	0.947	1.110	0.5345
Age male	1.001	0.940	1.067	0.9634
Duration of infertility	1.073	0.945	1.220	0.2775
Blastocyst	1.130	1.033	1.236	0.0076

**Table 3:** Demographic data of the no result embryos and the control group.

	Control group *N* = 1242	No result embryo group *N* = 93	*p* value
Female age	31.33 ± 6.14	32.62 ± 6.18	0.0609
Male age	34.89 ± 8.06	35.47 ± 7.87	0.3886
Duration of the infertility in years	2.114 ± 1.50	2.853 ± 2.24	0.0091
Cause of infertility			0.4501
Female factor	223 (18.0%)	15 (16.1%)	
Male factor	482 (38.8%)	29 (31.2%)	
Mixed	358 (28.8%)	39 (41.9%)	
Unexplained	179 (14.4%)	10 (10.8%)	
Consanguinity			0.5276
None	786 (63.7%)	52 (57.1%)	
Yes	448 (36.3%)	39 (42.9%)	
Subdivision of infertility causes			0.9188
Abnormal semen analysis	653 (52.6%)	39 (41.9%)	
PCOS	218 (17.6%)	23 (24.7%)	
Low ovarian reserve	157 (12.6%)	16 (17.2%)	
Uterine factor	141 (11.4%)	10 (10.8%)	
Tubal factor	63 (5.1%)	4 (4.3%)	
Endometriosis	10 (0.8%)	1 (1.1%)	

*Note:* Categorical data are presented as *N* (%). Data are analyzed by chi-square statistics to compare study groups. *p* value less than 0.05 is considered significant.

**Table 4:** Blastocyst characteristics in the control and the no result group.

	Control *N* = 1242	No result *N* = 93	*p* value
Day of biopsy			0.3718
Day 5	641 (51.6%)	45 (48.4%)	
Day 6	584 (47.0%)	45 (48.4%)	
Day 7	17 (1.4%)	3 (3.2%)	
Degree of expansion			0.0624
3	339 (27.3%)	34 (36.6%)	
4	100 (8.1%)	9 (9.7%)	
5	753 (60.6%)	45 (48.4%)	
6	50 (4.0%)	5 (5.4%)	
Grade at biopsy			0.0584
Excellent	554 (44.6%)	42 (45.2%)	
Good	246 (19.8%)	20 (21.5%)	
Average	316 (25.4%)	17 (18.3%)	
Poor	126 (10.1%)	14 (5.1%)	
Trophectoderm quality			0.3503
A	671 (54.0%)	55 (59.1%)	
B	556 (44.8%)	37 (39.8%)	
C	15 (1.2%)	1 (1.1%)	

*Note:* Categorical data are presented as *N* (%). Data are analyzed by chi-square statistics to compare study groups. *p* value less than 0.05 is considered significant.

**Table 5:** Regression analysis for the causative factors.

	OR	95% CI	*p* value
Number of oocytes	1.006	(0.978–1.035)	0.674
Number of blasts	0.923	(0.86–0.99)	0.026
Day of biopsy			0.71
Day 5	0.567	(0.1420.152)	0.42
Day 6	0.573	(0.152–2.163)	0.411
Grade of embryo			0.525
Excellent	0.434	(0.125–1.507)	0.189
Good	0.586	(0.21–1.637)	0.308
Average	0.603	(0.239–1.519)	0.283
Degree of expansion			0.480
3	0.865	(0.275–2.719)	0.804
4	0.880	(0.264–2.936)	0.836
5	0.618	(0.219–1.745)	0.363
Trophectoderm quality			0.320
A	2.831	(0.268–29.966)	0.387
B	1.357	(0.156–11.792)	0.782

*Note:* Variables entered on step 1 of the regression analysis: number of oocytes, number of blasts, day of biopsy, grade at the biopsy, degree of expansion, and trophectoderm quality. *p* values less than 0.05 are statistically significant.

## Data Availability

The datasets used and/or analyzed during the current study are available from the corresponding author on reasonable request.

## Supporting Information

Supporting Table 1: cycle characteristics of the no result group and the control group.
