# Supplementary material for: Embryos With “No Result” After PGT-A: A Retrospective Analysis of Causative Factors
Source: Obstet Gynecol Int. 2025 Mar 24;2025:4043963. doi: 10.1155/ogi/4043963 (PMC11957852; doi:10.1155/ogi/4043963)
Supplement: Supporting Information — Additional supporting information can be found online in the Supporting Information section. [file 4043963.f1.docx]

Supplementary Table 1: Cycle characteristics of the no result group and the control group

|  | Control  N=1242 | No result  N=93 | p-value |
| --- | --- | --- | --- |
| No. of oocytes | 24.09 ±11.11 | 21.69 ±11.5 | 0.041 |
| No. of mature oocytes | 19.53 ±9.08 | 17.17 ±9.03 | 0.024 |
| No. of fertilized oocytes | 16.53 ±7.57 | 14.44 ±7.43 | 0.016 |
| No. of blasts | 9.424 ±5.05 | 7.753 ±4.15 | 0.004 |
